# Intramuscular Injection of AAV8 in Mice and Macaques Is Associated with Substantial Hepatic Targeting and Transgene Expression

**DOI:** 10.1371/journal.pone.0112268

**Published:** 2014-11-13

**Authors:** Jenny A. Greig, Hui Peng, Jason Ohlstein, C. Angelica Medina-Jaszek, Omua Ahonkhai, Anne Mentzinger, Rebecca L. Grant, Soumitra Roy, Shu-Jen Chen, Peter Bell, Anna P. Tretiakova, James M. Wilson

**Affiliations:** Gene Therapy Program, Department of Pathology and Laboratory Medicine, Division of Transfusion Medicine, University of Pennsylvania, TRL Suite 2000, 125 South 31^st^ Street, Philadelphia, PA, 19104, United States of America; Justus-Liebig-University Giessen, Germany

## Abstract

Intramuscular (IM) administration of adeno-associated viral (AAV) vectors has entered the early stages of clinical development with some success, including the first approved gene therapy product in the West called Glybera. In preparation for broader clinical development of IM AAV vector gene therapy, we conducted detailed pre-clinical studies in mice and macaques evaluating aspects of delivery that could affect performance. We found that following IM administration of AAV8 vectors in mice, a portion of the vector reached the liver and hepatic gene expression contributed significantly to total expression of secreted transgenes. The contribution from liver could be controlled by altering injection volume and by the use of traditional (promoter) and non-traditional (tissue-specific microRNA target sites) expression control elements. Hepatic distribution of vector following IM injection was also noted in rhesus macaques. These pre-clinical data on AAV delivery should inform safe and efficient development of future AAV products.

## Introduction

Vectors derived from adeno-associated viruses (AAV) have been shown to produce long-term and stable gene expression of secreted proteins in a variety of animal models and human clinical trials following intramuscular (IM) injection, including coagulation factor IX (FIX) [1], [2], [3], [4], [5], alpha-1-antitrypsin (AAT) [6], [7], erythropoietin [8], and neutralizing immunoglobulins against HIV [9], [10]. Intramuscular (IM) delivery of an AAV vector provides a quick, easy, non-invasive and safe route of administration, which can be routinely performed in virtually any setting. The most celebrated example of IM AAV gene therapy is the treatment of an inherited deficiency of lipoprotein lipase with the commercially approved product Glybera [11]. However, previous studies have identified some of the limitations of IM injections, whereby transduction is limited to cells around the needle tract area of the injection site in mice, nonhuman primates (NHP) and humans [3], [12], [13], [14], [15]. This has led to the practice of a large number of small volume IM injections to produce sufficient transgene expression [7]. For example, subjects enrolled in the high dose cohorts of the Phase II AAT clinical trial received one hundred 1.35 ml vector injections IM spread across ten sites or up to sixty 0.5 ml injections in clinical trials for Glybera [6], [11].

Local injection of vector into most tissues could lead to a percentage of the injected volume disseminating from the site of injection and being transported to other organs. We speculate that the larger the volume of an IM injection, potentially the greater fraction of the volume can be dispersed from the site of administration. Therefore, due to the natural or increased tropism of certain AAV vectors for the liver and the resulting liver transduction, a significant contribution to the total level of a secreted transgene protein may be contributed by the liver following IM administration. Also, distribution of vector beyond the muscle could have implications on the safety and immunogenicity of the treatment. It has been suggested that delivery of the vector to liver, either by design or inadvertently, could induce immunologic tolerance to the transgene product, thereby diminishing immune toxicity [16], [17], [18], [19], [20], [21], [22], [23], [24]. In contrast, IM administration of concentrated AAV vectors, resulting in high vector dose per injection site, has been linked to higher level antibody production against the secreted transgene product [4], [5].

An AAV product can be engineered to restrict the expression of transgenes following different systemic routes of administration, such as IM, which will lead to broad distribution of vector. The more traditional approach to overcome this problem is to drive expression of the transgene from a tissue-specific promoter, such as the muscle creatine kinase (tMCK) promoter for skeletal muscle expression [25] and the human thyroxine binding globulin (TBG) promoter for liver expression [26], [27]. Transgene expression can also be inhibited in certain organs by the incorporation of tissue-specific microRNA target sites [28], [29]. Interaction of microRNAs with their complementary target sites within the RNA-induced silencing complex can lead to inhibition of translation or degradation of mRNA [30], [31]. Incorporation of 3–6 copies of target sites for the liver-specific microRNA (miR) 122 or skeletal muscle-specific miR-206 in the 3′ UTR of an AAV vector has been previously shown to reduce liver and muscle gene expression, respectively [32], [33], [34], [35]. Therefore, transgene expression could be restricted from either liver by miR-122 or muscle by miR-206.

In the current study we have evaluated important aspects of AAV8 IM delivery, such as concentration and volume, and features of the expression cassette, such as tissue specificity, on safety and efficacy in mice and macaques.

## Results

### Substantial gene expression from liver following IM administration of AAV8 vectors in mice

**Figure 1:**
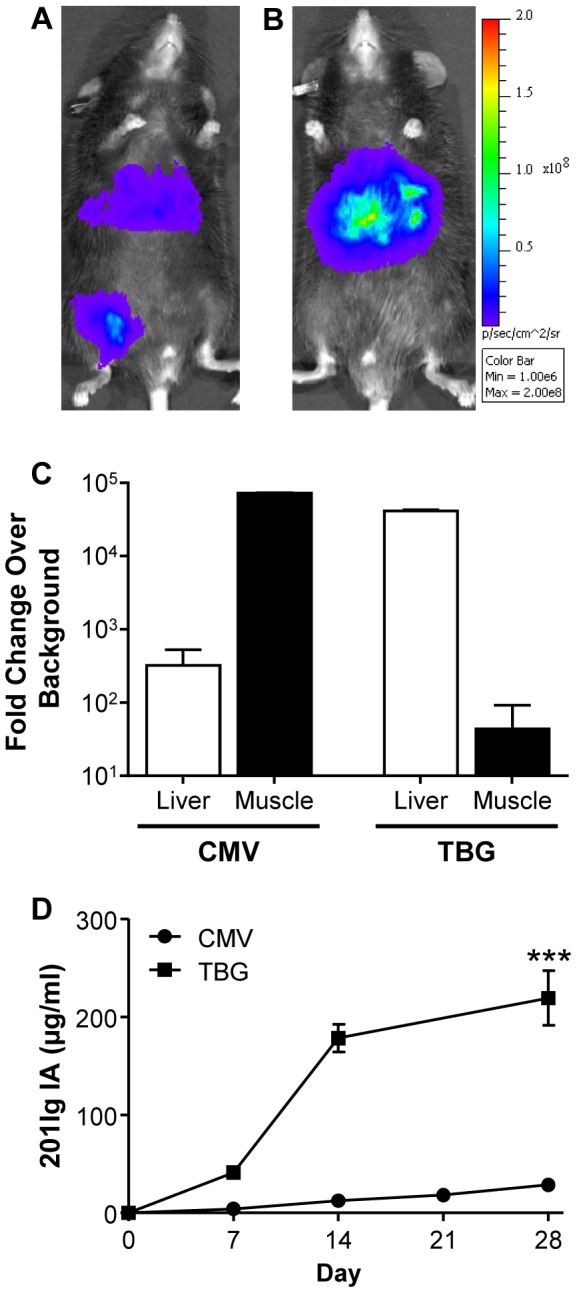
Liver expression following IM vector administration in mice. Visualization of differential ffLuc expression patterns using Xenogen whole-body bioluminescent imaging on day 7 post-IM administration of 10^10^ GC AAV8.CMV.ffLuc (A) or AAV8.TBG.ffLuc (B) to C57BL/6 mice. Vector was administered as one 10 µl injection into the gastrocnemius muscle of the right leg. (C) ffLuc expression was quantified at day 28 post-vector administration by ffLuc tissue assays. ffLuc expression measured as RLU normalized to the total protein concentration of the organ. Data are presented as fold change over background where background was RLU/total protein of organ (g) in control tissues from un-injected mice. (D) Comparison of expression of 201Ig IA from the CMV and TBG promoters following a 10 µl IM injection in RAG KO mice. Expression of 201Ig IA in serum was measured by ELISA. Values are expressed as mean ± SEM (n = 4/group). ***p<0.001.

AAV8 vector expressing firefly luciferase (ffLuc) from the ubiquitous CMV promoter was injected IM into C57BL/6 mice at a dose of 10^10^ genome copies (GC) per mouse (Figure 1A). Vector was administered as one 10 µl injection into the right gastrocnemius muscle and on day 7 post-vector mice were imaged to determine the localization of ffLuc expression. Significant ffLuc expression, which localized to both the injected muscle and the liver, was demonstrated (Figure 1A). These imaging results were quantitatively reproduced and further expanded using biochemical assays where ffLuc was measured in both liver and muscle samples and normalized to total protein on day 28 post-vector administration (Figure 1C). IM injection of vector using the CMV promoter for expression resulted in extensive ffLuc expression in the injected muscle with concordant expression in the liver, which was 321-fold over background (control un-injected mice).

The unexpectedly high levels of liver transduction after IM injection suggested that IM delivery could be an alternative to the standard way of targeting liver, which is by intravenous (IV) injection. Experiments were repeated with a vector expressing ffLuc from the highly potent, liver-specific promoter TBG [26], [27]. Mice were injected IM with 10^10^ GC of AAV8.TBG.ffLuc in a volume of 10 µl and at day 7 post-vector administration significantly higher liver expression was seen with little to no gene expression in muscle (Figure 1B). ffLuc tissue assays on liver and muscle taken at day 28 following administration of vector showed an increase in liver ffLuc expression of 69-fold, relative to that achieved with the CMV promoter (Figure 1C). While muscle ffLuc expression was over background (control un-injected mice) following IM injection, the three log reduction in muscle expression seen was expected due to the specificity of the TBG promoter (Figure 1C).

As ffLuc expression allowed tissue localization of gene expression, the net impact of inadvertent liver delivery of AAV following IM injection was studied by expression of a secreted protein. As antibodies expressed from AAV are being developed to prevent infections, including HIV and influenza [9], [10], [36], [37], these initial studies used a gene encoding 201Ig IA. This immunoadhesin (IA) construct was based on the 201Ig FAb, which was isolated from a long-term non-progressing rhesus macaque six years post-challenge with SIVsmF236 [38], [39]. RAG KO mice were injected IM with 10^10^ GC of AAV8 expressing 201Ig IA from the CMV or TBG promoters (Figure 1D). Immunodeficient RAG KO mice were used to evaluate expression of the 201Ig IA transgene in the absence of an immune response to the secreted protein. Substantial levels of IA expression were detected in blood with both vectors, although the liver-specific promoter yielded an almost 8-fold higher expression of the secreted protein in blood. This suggests that a substantial amount of the secreted IA is derived from liver, rather than muscle, after IM injection. Additional studies to evaluate the relative contribution of the two tissues/organs to blood levels of the transgene product are described below.

### Dose and route of administration impacts on expression in mice

**Figure 2:**
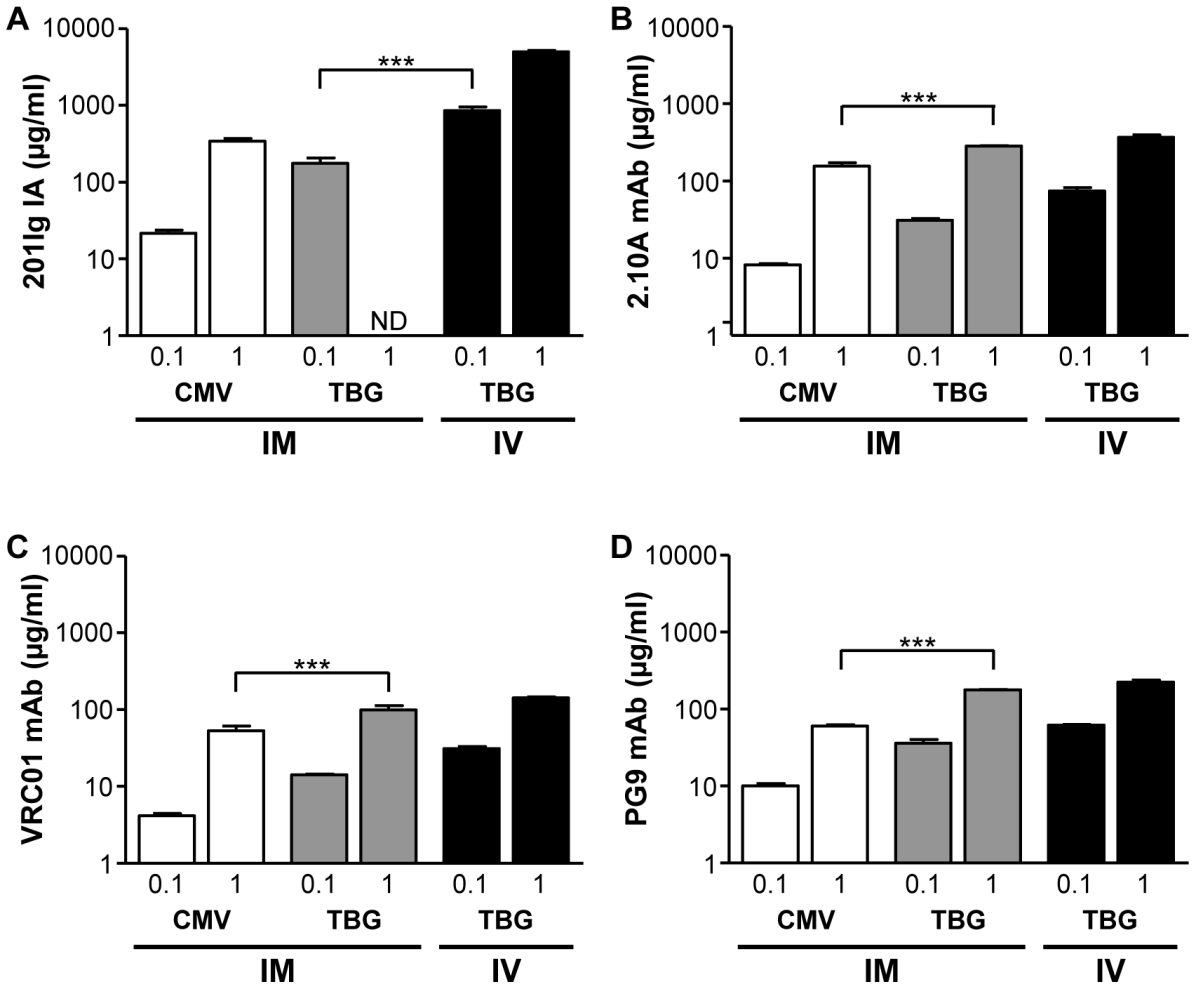
Comparison of expression from the liver-specific TBG promoter following IM and IV vector administration in RAG KO mice. Expression on day 28 post-vector administration of 201Ig IA (A), 2.10A mAb (B), VRC01 mAb (C), and PG9 mAb (D) in RAG KO mice. Expression following IM injection of vectors with either the CMV or TBG promoter was compared to expression levels produced following IV injection of the TBG vector. Mice were injected IM with two 15 µl injections into the right and left gastrocnemius muscles. IV injections were performed as a 100 µl injection via the tail vein. Mice were administered with a dose of either 10^10^ GC or 10^11^ GC, numbers indicate dose ×10^11^ GC. ND, not determined. Expression was measured in serum by ELISA and values are expressed as mean ± SEM (n = 3/group). ***p<0.001.

AAV8 vectors expressing anti-SIV/SHIV antibodies (in an immunoadhesin format [i.e., 201Ig IA] or a monoclonal antibody format [i.e., 2.10A mAb] [40]) or human anti-HIV antibodies in a monoclonal antibody format (i.e., VRC01 mAb [41], [42] and PG9 mAb [43]) were injected IM as two 15 µl injections at doses of 10^10^ GC or 10^11^ GC into RAG KO mice (Figure 2). Expression of these proteins in serum was determined on day 28 post-vector administration and compared to expression from TBG-containing vectors administered intravenously (IV) via the tail vein. Figure 2 summarizes data from these experiments, in which the two doses of vectors (labeled 0.1 and 1) were evaluated in the context of two comparisons: the CMV versus TBG vectors following IM injection and the TBG vector following IM and IV injection.

A dose-dependent increase in transgene expression was seen for all AAV8 vectors, independent of route of administration or promoter, where expression increased on average 8.5-fold across all transgenes when vector dose per mouse was increased by one log (Figure 2). Substantial differences were achieved when comparing expression from the CMV versus TBG promoter of vectors administered IM. A statistically significant increase in expression was observed with the TBG promoters, compared to the CMV promoters, at the highest dose of vector with the three mAb cassettes (Figures 2B-D). A direct comparison of IM versus IV injection of the TBG promoted vectors revealed little difference in expression of the antibodies at either dose of vector (Figures 2B-D). The exception to this was 201Ig IA, which was significantly increased (4.8-fold) following IV injection (Figure 2A). Therefore, a comparable level of secreted gene expression from a liver-specific promoter can be produced following a quick, easy and non-invasive injection into skeletal muscle or an invasive IV injection, which requires a higher level of skill.

### Modulation of liver and muscle gene expression by IM injection volume in mice

**Figure 3:**
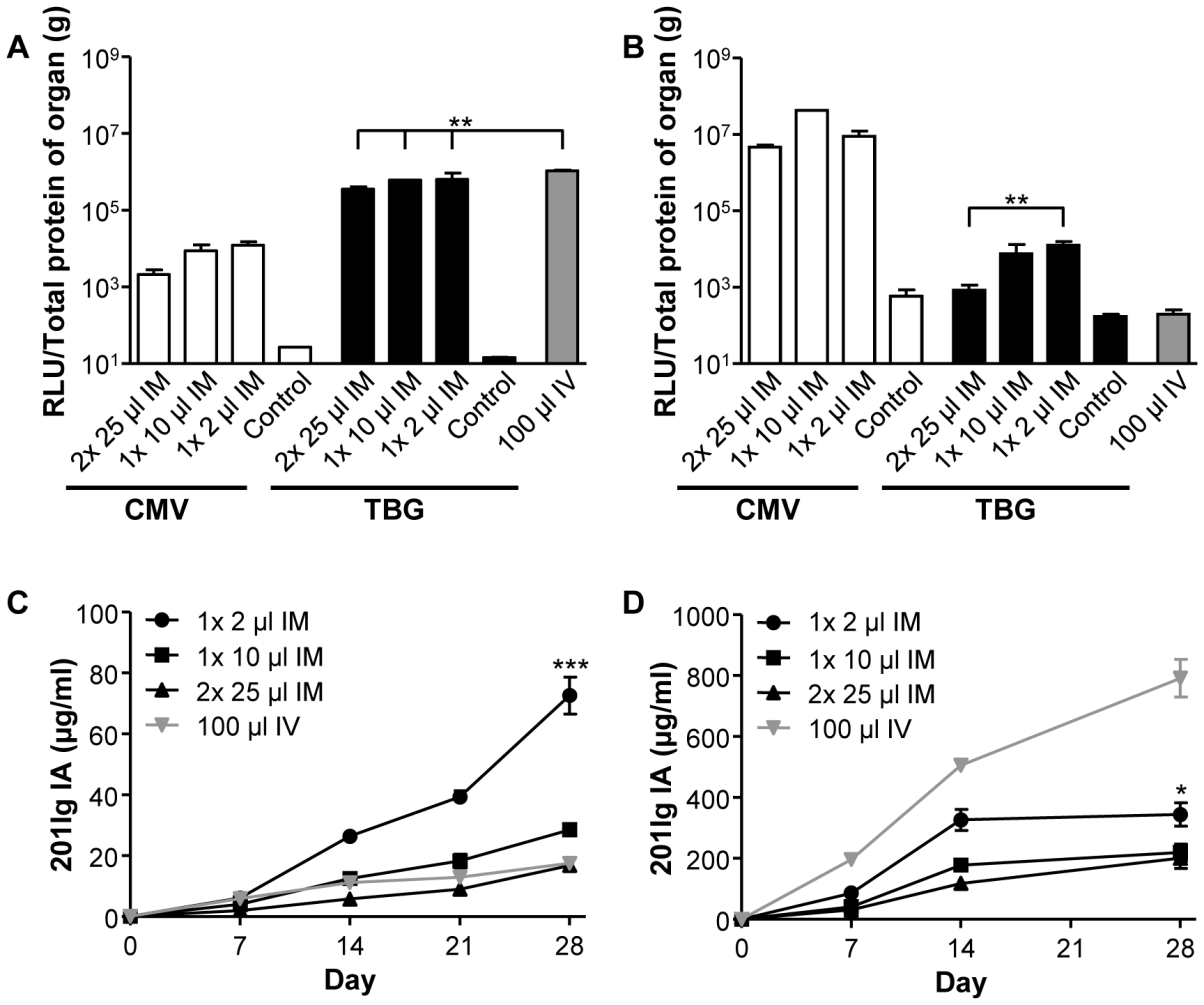
Reduction in IM injection volume increases transgene expression. 10^10^ GC AAV8 expressing ffLuc from the CMV or TBG promoter was delivered IM as either two 25 µl injections (one into each leg), one 10 µl injection or one 2 µl injection to C57BL/6 mice. IV injections were performed as a 100 µl injection via the tail vein. ffLuc tissue assays were performed on tissue harvested at day 28 and normalized to the total protein concentration of liver (A) and muscle (B). RAG KO mice were administered with 10^10^ GC AAV8.CMV.201Ig IA (C) or AAV8.TBG.201Ig IA (D) by IV or IM injections, performed as described previously. Data for the one 10 µl injection groups were previously presented in Figure 1. Expression of 201Ig IA in serum was measured by ELISA. Values are expressed as mean ± SEM (n = 4/group). *p<0.05, **p<0.01, ***p<0.001.

We investigated the impact of injection volume on the level and distribution of transgene expression. In these studies the same dose of vector was injected in a range of volumes from two 25 µl injections, one into each leg for a total injection volume of 50 µl, to one 2 µl injection in one site representing a 25-fold range of vector concentrations. The initial studies focused on mice injected with 10^10^ GC AAV8 vectors expressing ffLuc from the CMV or TBG promoters. Tissues were harvested at day 28 and transgene expression measured in lysates from liver (Figure 3A) and muscle (Figure 3B). Our original hypothesis was that increasing the volume for a fixed dose would increase the relative distribution of vector to liver. These studies did confirm the pilot experiments described in Figure 1, which used a single concentration of vector, whereby: 1) IM injection of vector resulted in substantial levels of transgene expression in liver, and 2) vectors using the TBG promoter produced levels of transgene product in liver following IM injection that were almost equivalent to the levels achieved when the same vector was injected IV. However, we were surprised to see that the more concentrated IM injections of vector did not help restrict expression to muscle; in fact these studies showed higher levels of liver ffLuc expression with lower volumes of vector. Also, the higher concentrations of vector yielded higher levels of overall ffLuc in both muscle and liver, independent of promoter. Based on *in vitro* experiments (data not shown), there was no significant loss of vector following dilution to different concentrations prior to administration in mice. Therefore, all mice received the same dose of vector and differences in distribution of the vector were due to the concentration of the injected vector.

The impact of IM injection volume was also studied in mice injected with AAV8 vectors expressing 201Ig IA from either the CMV (Figure 3C) or TBG promoter (Figure 3D) with the readout being serum levels of 201Ig IA. The CMV vectors (Figure 3C) showed dramatically lower overall expression as compared to the TBG vectors (Figure 3D). Serum 201Ig IA levels were not markedly affected by the volume of vector injected IM. Although the highest expression from the IM injected TBG vector was achieved with the lowest volume, it was still around two-fold lower than that achieved following IV injection.

**Figure 4:**
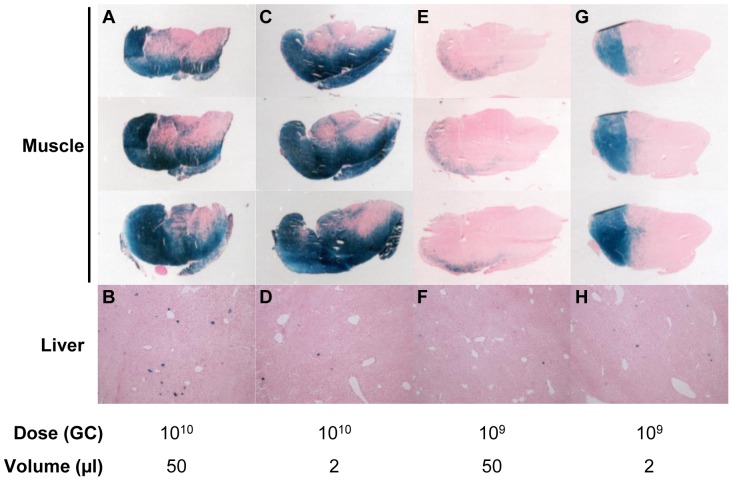
Localization of LacZ expression in injected muscle following IM injection of AAV8 in C57BL/6 mice. Localization of LacZ expression in injected muscle was determined at day 21 post-vector administration in C57BL/6 mice. Vector was administered at a dose of 10^10^ GC as two 25 µl injections into the right and left legs (A, B) or as one 2 µl injection (C, D). A lower dose of 10^9^ GC of vector was administered as two 25 µl injections into the right and left legs (E, F) or as one 2 µl injection (G, H). Three sections were taken throughout the injected gastrocnemius muscle of one representative animal per group (A, C, E and G) with one representative liver section per group (B, D, F and H) (n = 4/group).

A series of studies were performed using LacZ as a reporter gene to evaluate distribution of transduction at a cellular level as a function of vector concentration and dose. C57BL/6 mice were injected IM with AAV8 expressing LacZ from the CMV promoter. Liver and muscle tissue were harvested for analysis by LacZ histochemical staining 21 days post-vector administration (Figure 4). Sections of the gastrocnemius muscles were taken at intervals throughout the entire injected muscle. At a dose of 10^10^ GC, IM injection of vector as either two 25 µl injections (Figure 4A) or one 2 µl injection (Figure 4C) produced similar patterns of expression throughout the injected muscle, with a few transduced cells being seen in the liver (Figures 4B, 4D); note that the CMV promoter does not express well in liver. To determine if gene expression was saturated in the muscle at a dose of 10^10^ GC per mouse, the dose was lowered to 10^9^ GC per mouse and a very different transduction pattern was seen for the two injection volumes (Figures 4E, 4G). Injection of the vector IM as two 25 µl injections produced few transduced cells in the muscle (Figure 4E). When the concentration of the vector was increased to enable injection of the same dose of vector in a volume of 2 µl, a large area of transduced skeletal muscle cells were seen (Figure 4G). This transduced area extended through multiple muscle sections. Again, only a few transduced cells were seen in the liver at a vector dose of 10^9^ GC per mouse (Figures 4F, 4H). Therefore, administration of the same vector dose as a smaller injection volume improved skeletal muscle transduction at lower overall vector doses.

### Contributions of liver and muscle to total expression of a secreted transgene in mice

**Figure 5:**
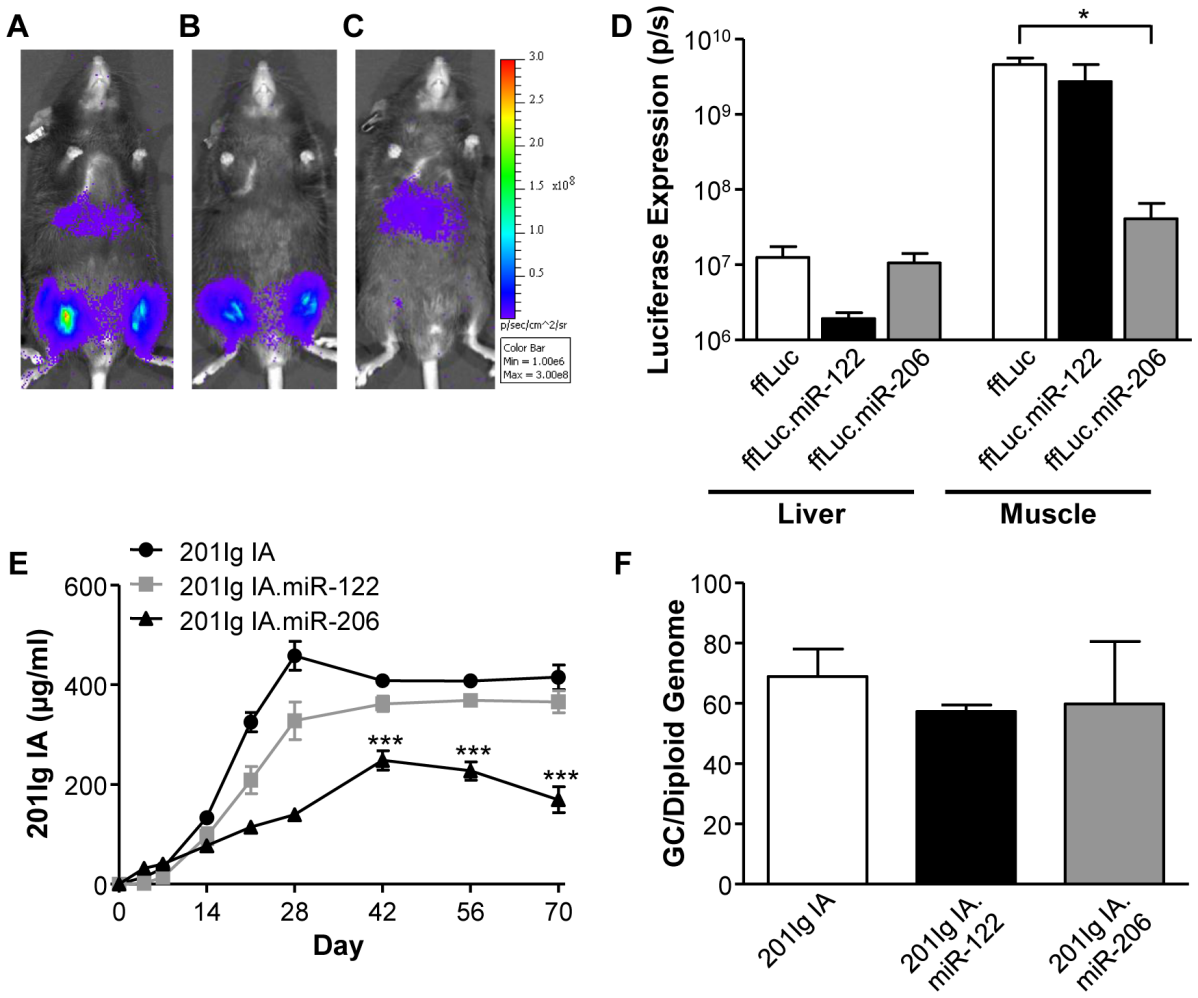
Effect of microRNA target sites on localization and expression level following IM injection of AAV8 in mice. Differential ffLuc expression patterns were visualized on day 7 post-IM administration of 10^10^ GC AAV8.CMV.ffLuc (A), AAV8.CMV.ffLuc.miR-122 (B) or AAV8.CMV.ffLuc.miR-206 (C). ffLuc expressing vectors were administered to C57BL/6 mice in two 10 µl injections into the right and left gastrocnemius muscles. (D) Liver and muscle expression of ffLuc was quantified separately on day 28 post-vector administration. Following substitution of ffLuc for 201Ig IA, expression of 201Ig IA was determined in RAG KO mice. Mice were injected with 10^11^ GC of vectors, administered as described previously and expression of 201Ig IA in serum was measured by ELISA (E). GC of the 201Ig IA vectors present in the liver of RAG KO mice at day 90 post-vector administration was determined (F). Values are expressed as mean ± SEM (n = 3/group). *p<0.05, ***p<0.001.

A critical issue not addressed in the experiments described above is the relative contribution of liver versus muscle in the production of secreted transgene products following IM injection of AAV8 vectors. Additional studies focused on vectors expressing the antibody 201Ig IA from a CMV promoter to simulate the likely clinical application of AAV expressed antibodies for prevention of infections, such as HIV and influenza [9], [10], [36], [37]. The strategy employed to address this issue utilized microRNA target sites that allow for tissue specific ablation of transgene expression. For example, the microRNA target sites for the liver-specific and the skeletal muscle-specific microRNAs, miR-122 and 206, respectively, have been previously shown to reduce gene expression in liver and muscle following the incorporation of these target sites within an AAV vector genome [32], [33], [34], [35]. Initial studies were performed with AAV8 vectors expressing ffLuc from a CMV promoter, with and without miR-122 and miR-206 target sites, in order to confirm the activity of the microRNA target sites. C57BL/6 mice injected IM with the ffLuc vectors were imaged for ffLuc expression on day 7 (Figures 5A-C) and expression was quantified on day 28 (Figure 5D). Incorporation of target sites for miR-122 and miR-206 into the 3′ UTR of the transgene produced specific knockdown of gene expression in the liver and muscle of 6.6- and 112-fold, respectively (Figures 5A-D). As previously demonstrated [32], [33], [35], [44], [45], [46], expression of the transgene in the organ that was not the target of microRNA-induced knockdown of gene expression remained unchanged (Figures 5A-D). These studies confirmed the usefulness of these microRNA target sites in specifically ablating liver or muscle expression.

Studies were conducted with vectors in which ffLuc was substituted with 201Ig IA in RAG KO mice, to evaluate expression in the absence of an immune response to the secreted protein. Expression of 201Ig IA following IM administration of an AAV8 vector containing the CMV promoter in the absence of miRNA target sites plateaued at 410 µg/ml on day 42 post-vector administration (Figure 5E). Restriction of transgene expression to muscle by the incorporation of miR-122 target sites reduced 201Ig IA expression, although not significantly, to 366 µg/ml (Figure 5E). In the presence of target sites for miR-206 to restrict expression to liver, 201Ig IA expression was significantly reduced to 169.2 µg/ml compared to the control vector (Figure 5E). GC analysis was performed for all groups and there was no significant difference in the number of GC present in the liver at day 90 post-vector administration, suggesting that the reduction in expression was due to the presence of the miRNA target sites and not ineffective vector administration (Figure 5F). These studies indicate that expression of a secreted antibody following IM injection of a CMV driven AAV8 vector in mice is primarily derived from muscle, although liver does contribute a substantial amount to total transgene expression.

### Hepatic distribution of AAV8 vector following IM injection is similar between mice and macaques

To help evaluate the relevance of these observations in mice to human clinical trials, we conducted comparison studies with rhesus macaques. In doing so we could not perform parallel studies of luciferase imaging since the size of the macaques precluded the use of this imaging modality. Therefore, we restricted our comparison studies to that of vector genome biodistribution.

**Figure 6:**
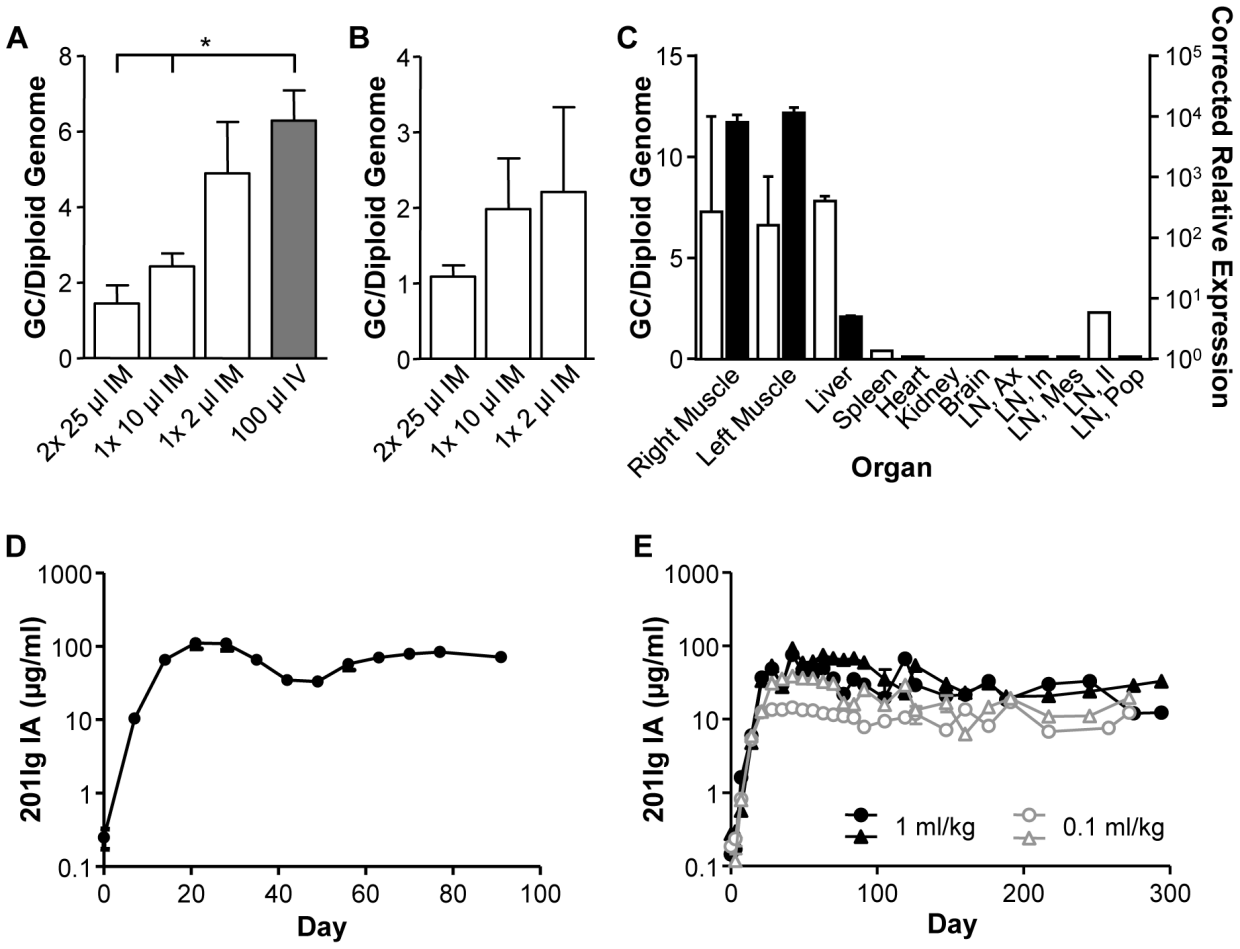
Translation of influence of injection volume to a large animal model, the rhesus macaque. RAG KO mice were injected with 10^10^ GC AAV8.CMV.201Ig IA either by IM or IV injection; tissues were harvested on day 56 and analyzed for vector genome copies (GC), quantified as GC/diploid genome in (A) liver and (B) muscle. (C) Biodistribution of AAV8 vector was determined on day 90 post-vector administration in a rhesus macaque. Vector was administered at a dose of 3×10^12^ GC/kg by IM injection into the vastus lateralis muscle of both right and left legs as 1 ml injections per kg body weight (vector concentration of 3×10^12^ GC/ml). DNA and RNA were extracted for quantification of GC (open bars) and transcript levels of 201Ig IA (closed bars), respectively. Values for muscle are the average of measurements at 12 sites throughout the injected muscle and liver is the average of the four lobes, which were quantified separately. There was no detectable GC or RNA in control (un-injected) muscle samples. LN, lymph node; Ax, axillary; In, inguinal; Mes, mesenteric; Il, iliac; Pop, popliteal. (D) Time course of expression of 201Ig IA in serum. (E) Rhesus macaques were injected IM with 3×10^11^ GC/kg of AAV8.CMV.201Ig IA, as either 1 ml vector injections per kg body weight (3×10^11^ GC/ml) or 0.1 ml injection per kg body weight (3×10^12^ GC/ml) (n = 2/group). Expression of 201Ig IA was measured in serum by ELISA and values are expressed as mean ± SEM. *p<0.05.

Mouse studies were performed with an AAV8 vector expressing 201Ig IA from a CMV promoter with tissues harvested at day 56. Animals were injected IM with 10^10^ GC of vector in different volumes (2×25 µl, 1×10 µl, and 1×2 µl). The deposition of vector significantly increased in liver as the concentration of the vector increased, consistent with the effects of vector concentration on transgene expression (Figure 6A). While there was not a significant increase in muscle, there was a trend towards higher GC following administration of smaller injection volumes (Figure 6B). The amount of vector present in the liver from the most concentrated dose delivered IM was close to, but slightly lower, than that observed following an IV injection of the same dose of vector (Figure 6A). Injection of vector IM in its most concentrated form yielded GC/diploid genome in muscle 2-fold lower than that in liver, again supporting substantial targeting of liver after IM injection of vector.

A male rhesus macaque was administered with the same vector at a dose of 3×10^12^ GC/kg by IM injection into the right and left vastus lateralis muscles, as 1 ml injections per kg body weight. This simulates the conditions of vector delivery in the mice in terms of concentration (NHP = 3×10^12^ GC/ml; mice = 5×10^12^ GC/ml with 2 µl injection and 1×10^12^ GC/ml with 10 µl injection), although the total dose of vector administered to mice was 10-fold lower than in NHPs. Figure 6C presents a biodistribution analysis of vector across a wide range of tissues, including muscle and liver. GC in the injected muscles are presented as the average of samples taken from 12 sampling sites within the muscle, which average 7.3 and 6.6 GC/diploid genome for the right and left injected muscles, respectively (Figure 6C). No GCs were detected in the control (un-injected bicep) muscle samples. GC for the four lobes of the liver were determined separately, the average of which was 7.8 GC/diploid genome (Figure 6C). GC biodistribution in the rhesus macaque were not qualitatively different from that in mice. GC in muscle and liver was higher in the macaque than in mice presumably reflecting the 10-fold higher dose of vector administered to the macaque, although the increase was higher in muscle (3-fold) than what was observed in liver (50%). 201Ig IA RNA levels in the injected muscles and liver were also determined (Figure 6C). Very high level transcription of the transgene was seen in the injected muscles with substantially lower corrected relative expression of 201Ig IA per GC seen in liver, possibly due to the reduced activity of the CMV promoter in liver. Serum levels of 201Ig IA were determined in the injected rhesus macaque and no drop in transgene expression was seen throughout the course of the study, indicating a lack of either neutralizing antibody (NAb) production to the transgene or destructive cytotoxic T lymphocyte response to the transgene expressing muscle (Figure 6D).

### Small volume vector administration by IM injection has no effect on transgene expression in rhesus macaques

To allow for translation of the effect of vector concentration on expression in mice to human subjects in gene therapy clinical trials, a large animal model was required for extrapolation of more relevant vector doses per injection site and per kg body weight. Rhesus macaques were administered IM with 3×10^11^ GC/kg of AAV8.CMV.201Ig IA with the vector being injected as either 1 ml vector injections per kg body weight (3×10^11^ GC/ml) or 0.1 ml per kg body weight (3×10^12^ GC/ml). There was no significant difference in 201Ig IA expression between the groups, which has remained constant for nearly one year post-vector administration (Figure 6E). Contrary to previous observations, for AAV8 vectors expressing 201Ig IA in rhesus macaques there appears to be no evidence of NAbs to the transgene being generated with high vector doses per injection site.

## Discussion

An important finding from our study is the extensive distribution of AAV8 vector to liver following IM injection in both mice and macaques. While this issue has indeed been raised as a consequence of muscle delivery in previous studies, we show that the hepatic deposited gene may contribute substantially to systemic production of transgene products, especially if the promoter is active in hepatocytes. These findings have relevance to both pre-clinical and clinical applications of gene therapy.

A number of studies have attempted to evaluate the performance of AAV-mediated gene transfer to skeletal muscle in the context of secreted transgene products. We show, using microRNA target sites to selectively ablate liver versus muscle expression, that with AAV8 vectors containing the more muscle-specific CMV promoter, the majority of transgene product in the blood is derived from transduced muscle with liver contributing to at least one third of the total product. Liver dominated the production of the secreted transgene product following IM injections with a vector containing a potent and liver-specific promoter. Therefore, one may erroneously ascribe vector performance to muscle transduction following IM injection in situations with capsids that target liver and with promoters that have activity in both muscle and liver, such as the frequently used CMV-enhanced β-actin (CAG) promoter.

The inadvertent targeting of liver following IM injection of skeletal or cardiac muscle has been seen as a safety concern and has led to strategies to “de-target” liver through capsid engineering or restricting transcription. A series of studies have emerged that suggest some delivery of vector to the liver may actually be beneficial in terms of avoiding immune toxicity. Herzog and colleagues were the first to show that IV delivery of AAV was associated with induction of tolerance via the activation of transgene product specific regulatory T cells, which is consistent with the previously known tolerogenic nature of the liver [16], [17], [18], [19], [20], [21], [22], [23], [24]. AAV induced tolerance is being considered as a way to avoid immune responses in protein replacement therapies. The properties of the AAV capsid will also influence transgene immunogenicity. We previously showed in hemophilia B mice that IM injection of hepatotropic vectors, such as AAV8, avoided factor IX antibodies, which was not the case with vectors that poorly transduce liver, such as AAV1 and AAV5 [47].

Our studies also demonstrate the impact of vector dose and concentration on efficiency of muscle transduction and distribution to liver. Efficient muscle transduction occurred at high dose of vector, independent of concentration, although this was not the case when the overall dose of vector was decreased, at which point more concentrated injections achieved higher muscle transduction. The highest concentration of injected vector in our study was 5×10^12^ GC/ml in mice and 3×10^12^ GC/ml in rhesus macaques, which is essentially identical to the concentration of vector that was injected in several AAV IM clinical studies including hemophilia B, AAT deficiency, and the commercially approved product Glybera [6], [7], [11], [48]. In these studies, research subjects received up to 60–100 injections at the highest dose. Previously, attempts to increase concentration in order to decrease the number of injections were hampered by the limit of AAV solubility for most capsids in standard formulations being thought to be 10^13^ GC/ml. However, recently it has been reported that AAV8 vectors can be concentrated up to 10^14^ GC/ml [49]. Additionally, low vector dose per site was reported to reduce the potential for transgene-specific neutralizing antibodies following IM administration of AAV vectors expressing cFIX in dogs [4], [5]. These studies had utilized AAV1 and AAV2 vectors and their differences in hepatotropism versus AAV8 could provide explanation for the lack of evidence for this effect with AAV8.

Our studies indicate that expression does not suffer, and in fact may be improved, with higher concentrations of injected vector, which suggests that more efficient transduction through capsid engineering and/or optimized expression cassettes will likely yield more production of the secreted transgene product. There clearly will be a limit to how much transgene product can be produced from a muscle cell, at which time protein biogenesis pathways will be saturated and immune responses may be enhanced. In conclusion, these pre-clinical data for delivery of AAV via IM administration should inform on the safe and efficient development of future AAV gene therapy products.

## Materials and Methods

### AAV vector production

All AAV vectors were produced by the Penn Vector Core at the University of Pennsylvania as described previously [50]. Briefly, plasmids expressing firefly luciferase (ffLuc) or antibody constructs driven by the cytomegalovirus (CMV) or human thyroxine binding globulin (TBG) promoter were packaged with the AAV8 viral capsid. ffLuc and 201Ig IA plasmids driven by the CMV promoter containing microRNA target sites were produced by insertion of six copies of either the miR-122 or miR-206 target sites into the 3′ UTR region. Target sites were synthesized separated by restriction enzyme recognition sites, indicated by the upper case letters. The microRNA target site sequences were as follows: 6xmiR-122; TCTAGAcaaacaccattgtcacactccaGGATCCcaaacaccattgtcacactccaCGTACGcaaacaccattgtcacactccaACGCGTcaaacaccattgtcacactccaCACGTGcaaacaccattgtcacactccaGCATGCcaaacaccattgtcacactccaGCGGCCGC, 6xmiR-206; TCTAGAccacacacttccttacattccaGGATCCccacacacttccttacattccaCGTACGccacacacttccttacattccaACGCGTccacacacttccttacattccaCACGTGccacacacttccttacattccaGCATGCccacacacttccttacattccaGCGGCCGC.

### Mice

Male C57BL/6 and RAG KO mice at 6–8 weeks of age were purchased from Charles River Laboratories (Wilmington, MA, USA) and The Jackson Laboratory (Bar Harbor, ME, USA), respectively. Mice were housed under specific pathogen-free conditions at the University of Pennsylvania's Translational Research Laboratories. All animal procedures and protocols were approved by the Institutional Animal Care and Use Committee (IACUC, approval number 803395) of the University of Pennsylvania. Mice were sacrificed by carbon dioxide asphyxiation and death was confirmed by cervical dislocation.

Mice were anaesthetized with a mixture of 70 mg/kg of body weight ketamine and 7 mg/kg of body weight xylazine by intraperitoneal (IP) injection for all IM injections. Vector was diluted in phosphate buffered saline (PBS) and IM injections were performed using a Hamilton syringe. IV injections of vector were performed by injection of 100 µl vector dilution into the tail vein. Serum was collected weekly from mice administered with vectors expressing secreted proteins by retro-orbital bleeds into serum collection tubes.

### NHP

Rhesus macaques (Chinese origin and captive bred, 3.75–4.15 kg) were housed at the Nonhuman Primate Research Program facility of the Gene Therapy Program of the University of Pennsylvania (Philadelphia, PA) during the studies. Studies were performed according to a study protocol approved by the IACUC (approval number 804625), the Environmental Health and Radiation Safety Office and the Institutional Biosafety Committee of the University of Pennsylvania. All animals were housed in stainless steel caging with perches and maintained on a 12-hour light/dark cycle controlled via an Edstrom Watchdog system. Temperature was maintained within the range of 18–26°C with 50% (±10%) humidity. Animals were fed Certified Primate Diet 5048 (PMI Feeds Inc., Brentwood, MO, USA) two times per day (morning and evening). Water was available *ad libitum* from an automatic watering system. Food enrichment such as fruits, vegetables, nuts and cereals were provided daily. Manipulanda such as kongs, mirrors, puzzle feeder and raisin balls were provided daily. Animals also received visual enrichment along with human interaction on a daily basis.

All macaques had NAb titers of <1:5 at the start of the studies determined as described previously [51]. Prior to vector administration, macaques were anesthetized with a mixture of ketamine (10–15 mg/kg) and dexmedetomidine (0.05–0.10 mg/kg) injected IM into the bicep muscle. One macaque was administered with a dose of 3×10^12^ GC/kg AAV8.CMV.201Ig IA into the vastus lateralis muscle of both right and left legs as 1 ml injections per kg body weight (vector concentration of 3×10^12^ GC/ml) for the vector biodistribution study. To study the influence of vector dose per injection site, rhesus macaques were administered with a dose of 3×10^11^ GC/kg as 1 ml injections per kg body weight (vector concentration of 3×10^11^ GC/ml) into the vastus lateralis muscle of both right and left legs or administered with the same vector dose as 0.1 ml injection per kg body weight (vector concentration of 3×10^12^ GC/ml) into the right vastus lateralis muscle. Blood samples were taken pre-study and weekly during the study via venipuncture of the femoral vein. Clinical pathology was conducted by Antech Diagnostics (Irvine, CA, USA), including complete blood counts and differentials, and complete clinical chemistries, and transgene expression levels were measured in serum by ELISA.

On day 90 post-vector administration the rhesus macaque that received a dose of 3×10^12^ GC/kg was euthanized. The animal was first anesthetized as described previously and euthanized using sodium pentobarbital (80 mg/kg) injected IV. Death was confirmed by absence of heartbeat and respiration.

### Imaging

ffLuc expression was visualized by whole-body bioluminescence imaging weekly. Mice were injected IP with 10 µl/g body weight of 15 mg/ml luciferin (XenoLight D-Luciferin Potassium Salt; Perkin Elmer, Waltham, MA, USA). Five minutes post-luciferin injection, mice were anaesthetized with ketamine/xylazine and 15 minutes post-luciferin injection mice were imaged using IVIS Xenogen imaging system (Perkin Elmer, Waltham, MA, USA). ffLuc expression was quantified using regions of interests in Living Image 3.0 Software (Perkin Elmer, Waltham, MA, USA) and measured in photons per second (p/s).

### ffLuc Tissue Assay

Total injected muscle and a sample of liver were taken from mice at the time of necropsy. Tissue samples were frozen on dry ice and stored at −80°C. On the day of assay, tissue was weighed and then homogenized in 500 µl 1x Luciferase Cell Culture Lysis Reagent (CCLR; Promega, Madison, WI, USA) using the QIAGEN TissueLyser (Valencia, CA, USA). Samples were then centrifuged for 3 min at 10,000 x g at 4°C. The supernatant was assayed following the Luciferase Assay System Protocol (Promega, Madison, WI, USA). ffLuc tissue assay values were normalized to total protein of the origin organ, either muscle or liver. A BCA assay was performed on all supernatants from the ffLuc tissue assay, diluted 1∶1000 in PBS and using the Pierce BCA Protein Assay Kit (Thermo Scientific, Waltham, MA, USA). Total protein values were determined by the following calculation: (protein concentration of tissue sample by BCA/weight of tissue sample homogenized) x average weight of whole organ.

### Detection of secreted proteins in mouse serum

Antibody constructs were measured by ELISA where all reagents were from Sigma-Aldrich (St. Louis, MO, USA) unless otherwise stated. ELISA plates were coated with 5 µg/ml protein A, diluted in PBS and incubated overnight at 4°C. Wells were washed eight times with 0.05% Tween 20 in PBS and blocked with 1% BSA in PBS for one hour at room temperature. Following removal of the blocking buffer, heat inactivated serum samples diluted in PBS were added to the plates and incubated at 37°C for one hour. Plates were then washed eight times, blocked as described previously and Bio-SP-conjugated Affinipures Goat Anti-Human IgG antibody (Jackson ImmunoResearch Laboratories, Inc., West Grove, PA, USA) was added at a 1∶10,000 dilution. Following incubation at room temperature for one hour, plates were washed eight times and strepdavidin protein conjugated to horseradish peroxidase (HRP) was added at a 1∶30,000 dilution. After another incubation at room temperature for one hour, plates were washed eight times and 3,3′,5,5′-tetramethylbenzidine (TMB) was added for detection. The reaction was stopped after 30 minutes at room temperature using 2N sulfuric acid and plates were read at 450 nm using a BioTek µQuant plate reader (Winooski, VT, USA).

### Detection of secreted proteins in NHP serum

Detection of 201Ig IA in NHP serum was measured by antigen-specific capture ELISA. Following a similar procedure as that described above, ELISA plates were coated with 2 µg/ml SIVmac251 gp120 protein diluted in PBS and incubated overnight at 4°C. Wells were washed five times and blocked with 1 mM EDTA, 5% heat inactivated PBS, 0.07% Tween 20 in PBS for one hour at room temperature. All subsequent steps were the same as described previously.

### LacZ staining

LacZ gene expression was examined 21 days after vector administration by methods described previously [52].

### Vector biodistribution

Liver and muscle samples were snap frozen at the time of necropsy and DNA was extracted using the QIAamp DNA Mini Kit (Qiagen, Valencia, CA, USA). Detection and quantification of vector genomes copies (GC) in extracted DNA were performed by real-time PCR as described previously [53]. Briefly, genomic DNA was isolated and vector GCs were quantified using primers/probe designed against the SV40 poly adenylation sequence of the vector. Quantification of GC from liver was performed on one liver sample from each mouse (n = 4/group) and on a sample from each of the lobes of the liver in the NHP, data is presented as an average of the lobes. DNA extraction was performed on a homogenate of the entire gastrocnemius muscle from mice or from samples taken at 12 sampling sites throughout the injected vastus lateralis muscles and control bicep muscle from NHP. The average of the 12 samples per muscle is presented.

### RNA isolation and real-time PCR

RNA was isolated from NHP muscle and liver samples using TRIZOL (Life Technologies, Carlsbad, CA, USA) according to the manufacturer's protocol. 10 µg of RNA was then treated with DNase I (Roche, Basel, Switzerland) according to the manufacturer's protocol. The RNeasy Mini Kit (Qiagen, Valencia, CA, USA) was used to remove DNase prior to cDNA synthesis by reverse transcription using the Applied Biosystems High Capacity cDNA Reverse Transcriptase Kit (Life Technologies, Carlsbad, CA, USA). Real-time PCR was then performed on cDNA with primers binding to the 201Ig IA transgene with Power SYBR Master Mix for detection or primer/probe set for 18S with TaqMan Gene Expression Master Mix for detection (Life Technologies, Carlsbad, CA, USA). Relative transcript expression was determined using the ΔΔCT of each sample normalized to 18S expression.

### Statistical analysis

All analyses were performed in Prism (GraphPad Software, San Diego, CA, USA). A *p* value of <0.05 was considered significant. Comparisons between two groups were performed using unpaired Student's t-test and comparisons between multiple groups were performed using one-way analysis of variance (ANOVA, Tukey's Multiple Comparison post-test). All values expressed as mean ± standard error of the mean (SEM).
